# Polymeric Ionic Liquid‐Enabled *In Situ* Protection of Li Anodes for High‐Performance Li‐O_2_ Batteries

**DOI:** 10.1002/cssc.202402102

**Published:** 2024-11-28

**Authors:** Dan Li, Qian Chen, Rui Li, Yaolin Hou, Yulong Liu, Haiming Xie, Jia Liu, Jiefang Zhu

**Affiliations:** ^1^ Nation & Local United Engineering Laboratory for Power Batteries Faculty of Chemistry Northeast Normal University Changchun Jilin 130024 China; ^2^ Department of Chemistry – Ångström Laboratory Uppsala University SE-751 21 Uppsala Sweden; ^3^ Jilin Province Dongchi New Energy Technology Co. Ltd. Changchun Jilin 130000 China; ^4^ The Key Laboratory for Ultrafine Materials of The Ministry of Education East China University of Science and Technology Shanghai 200237 China

**Keywords:** Li-O_2_Batteries, Interface evolution, Synchrotron X-ray tomography, shuttle effect, Li anode protection

## Abstract

Redox mediators (RMs) have shown promise in enhancing Li‐O_2_ battery cycling stability by reducing overpotential. However, their application is hindered by the shuttle effect, leading to RM loss and Li anode corrosion. Here, we introduce a polyionic liquid, poly (1‐Butyl‐3‐vinylimidazolium bis(trifluoromethanesulfonylimine)) ([PBVIm]‐TFSI) as an additive, showcasing a novel Li anode protection strategy for LiI‐mediated Li‐O_2_ batteries. [PBVIm]^+^ cations migrate to the Li anode, forming a protective cationic shield that promotes uniform Li^+^ deposition. The addition of [PBVIm]‐TFSI enhances the cycling stability, achieving 105 cycles at 200 mA⋅g^−1^, compared to the cell with LiI which exhibited 38 cycles under the same conditions. Synchrotron X‐ray tomography reveals the evolution of this protective layer, providing insights into its formation mechanism, in conjunction with XPS analysis. Our findings offer a new approach to Li anode protection in Li‐O_2_ batteries, emphasizing the critical role of interfacial engineering for battery performance.

## Graphical abstract

The incorporation of the polyionic liquid additive [PBVIm]‐TFSI in LiI‐mediated Li‐O_2_ batteries enables the *in situ* formation of a protective layer on the Li anode. [PBVIm]^+^ cations migrate to the Li anode, forming a cationic shield that promotes uniform Li^+^ deposition. A [PBVIm][TFSI]‐containing cell demonstrates a considerable reversibility and cycling stability.

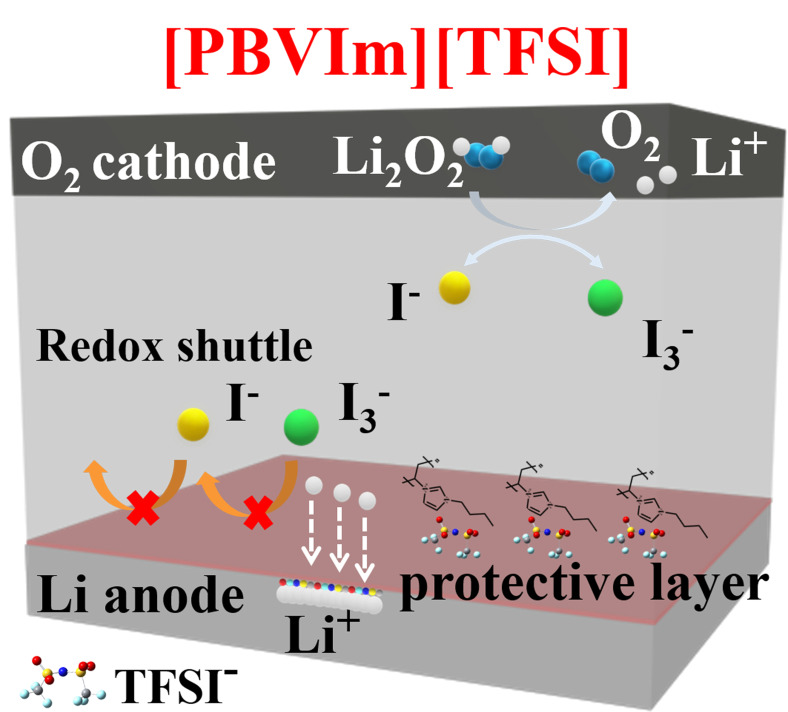

## 1 Introduction

The growing demand for high‐performance energy storage devices has led to increased interest in lithium‐oxygen (Li‐O_2_) batteries due to their high theoretical energy densities.[^1^, ^2^] However, the sluggish reaction kinetics during the formation and decomposition of Li_2_O_2_ pose significant challenges, resulting in low round‐trip efficiency and poor cyclability.[^3^, ^4^, ^5^] Redox mediators (RMs) have shown promise in enhancing the cycling stability of Li‐O_2_ batteries by reducing overpotential. These RMs tune electrochemical reactions from a surface adsorption pathway to a solvation‐mediated pathway.[^6^, ^7^] Unfortunately, the diffusion of soluble RM^−^/RM/RM^+^ species towards the Li anode, known as the redox shuttling effect, leads to undesired side reactions.^[8]^ This problem causes RM loss and continuous deterioration of the Li anode, ultimately decreasing the cycling stability.^[9]^ Therefore, new strategies are needed to reduce or suppress this shuttle effect in RM‐based Li‐O_2_ batteries.^[10]^

Various approaches have been explored to suppress the redox shuttling effect, including the development of modified separators, solid‐state electrolytes, artificial protective layers on the Li anode, and the introduction of electrolyte additives.[^11^, ^12^] Among these strategies, electrolyte additives offer several advantages, such as ease of operation, cost‐effectiveness, and high yield.^[13]^ Commonly used electrolyte additives include lithium salts, inorganic compounds, and organic compounds.^[14]^ However, lithium salt additives, such as LiF, LiNO_3_, and LiDFOB, are costly and limited in variety.^[15]^ Inorganic compound additives may lead to cycling instability due to subtle changes in size, morphology, and crystal phase. In contrast, ionic liquids (ILs), particularly pyrrole‐ and imidazolyl‐based ones, have gained extensive attentions as organic additives due to their low cost, renewability, and self‐repairing properties. In addition, the ease of IL arises from the vast range of available cations and anions, which can be paired to obtain the final products with the various properties including polarity, viscosity, hydrophobicity, ion conductivity, electrochemical stability, *etc*. This can meet numbers of application requirements *via* the custom‐tailored designs.[^16^, ^17^, ^18^, ^19^, ^20^] Several studies have demonstrated the potential of ILs in improving the performance of Li‐O_2_ batteries. For example, Freunberger *et al*., showed that DABCOnium, an IL derived from the monoalkylation of 1,4‐diazabicyclo[2.2.2]octane (DABCO), acted as a highly efficient singlet oxygen (^1^O_2_) quencher and contributed to reducing parasitic reactions.^[21]^ Yoon *et al*., employed ammonium‐based IL‐functionalized phenothiazine (IL‐PTZ) In Li‐O_2_ batteries, attributing the inhibition of side reactions between RM and Li metal by forming a solid electrolyte interphase (SEI) on the Li anode, thereby promoting cycling stability.^[22]^ In another study, Wang *et al*., used a 2,2,6,6‐tetramethyl‐1‐piperidinyloxy moiety (IL‐TEMPO) as a multi‐functional agent, which remarkably improved cycling performance by facilitating ORR/OER kinetics, protecting the Li anode, and suppressing side reactions.[^23^, ^24^] Polymeric ionic liquids (PILs), synthesized by polymerizing IL monomers with the assistance of an initiator, combine the desirable properties of ILs with the binding ability of polymers. Additionally, PILs offer several distinctive benefits due to their polymeric structures, which is not achievable with either inorganic additives or ILs. For example, compared to inorganic additives, PILs display more uniform and stable electrochemical stabilities with little risk of particle agglomerations, crystal structure changes, and the corresponding side reactions. Equally, PILs offer bigger mechanical strengths and structural integrities than those of ILs, which is beneficial to the electrochemical reactions. Currently, PILs have demonstrated their effectiveness in improving the cycling performance of Li‐ion batteries,[^25^, ^26^, ^27^] and have shown broad application prospects in anode protection for Li metal batteries, even Li‐O_2_ and Li‐S batteries.[^28^, ^29^, ^30^] However, reports on the utilization of PILs in RM‐based Li‐O_2_ batteries are very scarce, indicating a clear need for further investigation.

Herein, for the first time, we develop poly(1‐Butyl‐3‐vinylimidazolium bis(trifluoromethanesulfonylimine)) ([PBVIm]‐TFSI) as a PIL additive to enhance the cyclability and reversibility for LiI‐based Li‐O_2_ batteries. [PBVIm]^+^ can not only promote the uniform deposition of Li^+^ due to a cationic electrostatic shielding effect, but also facilitate the decomposition of Li salt to *in situ* generate a uniform protective layer on Li anode. This contributes to reducing the shuttle effect of I_3_^−^ and promoting the cycle life. Moreover, we employ synchrotron X‐ray computed tomography (SXCT) as an *in situ* approach to propose the inhibition mechanism for redox shuttling through tracking the morphology evolution of cell interfaces. Furthermore, the chemical composition evolution for Li anode during the deposition process is investigated by XPS depth profiling, thereby exploring the formation mechanism of the protective layer. Compared to a cell with only LiI, the one with extra [PBVIm]‐TFSI exhibits improved cycling stability, considerable rate performance, and good reversibility associated with Li_2_O_2_ formation and decomposition. This work provides valuable insights into the development of novel Li anode protection strategies for RM‐involved Li‐O_2_ batteries, emphasizing the critical role of interfacial engineering in enhancing overall battery performance.

## 2 Result and Discussion

**Figure 1:**
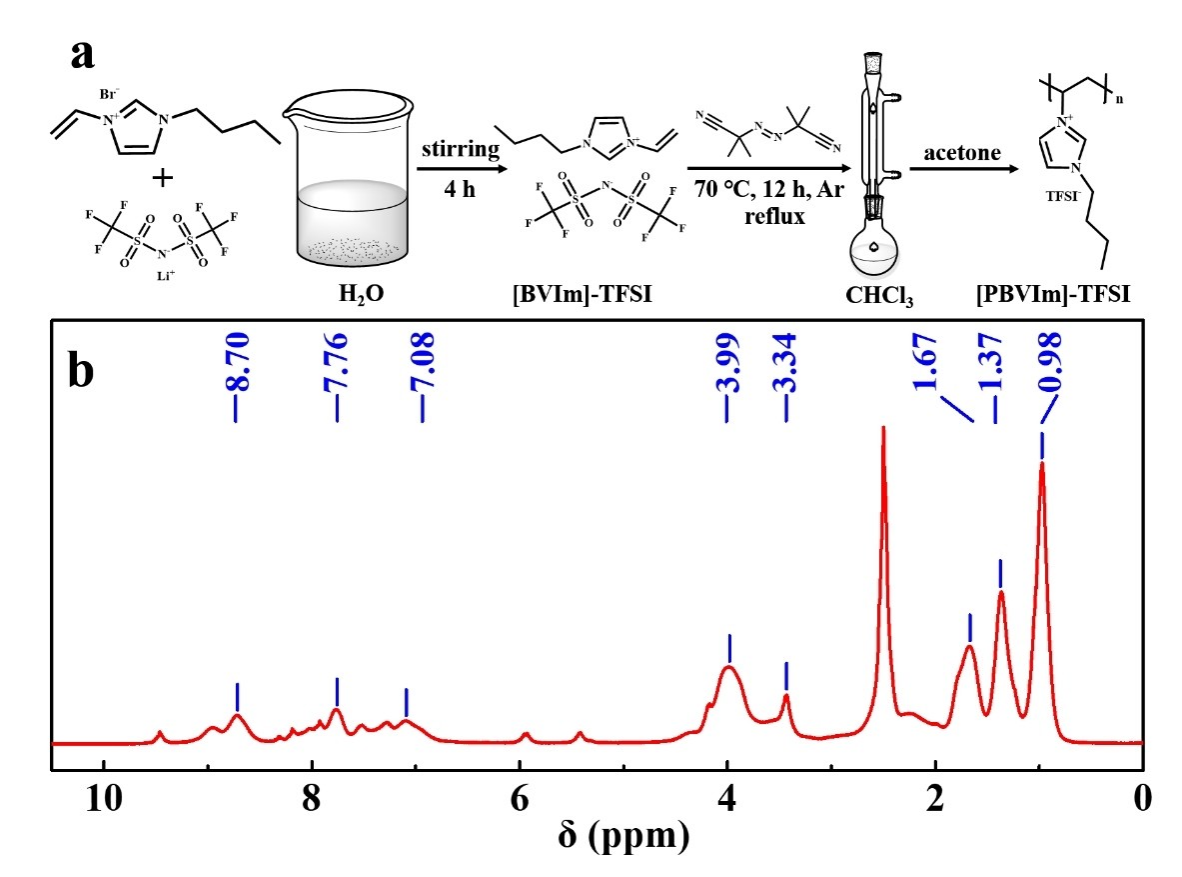
(a) Schematic of the synthesis for [PBVIm]‐TFSI, (b) ^1^H NMR spectrum of [PBVIm]‐TFSI complex in DMSO‐d6.

The synthesis process for [PBVIm]‐TFSI was described in the experimental section and shown in Figure 1a. In order to confirm the chemical structure for as‐prepared [PBVIm]‐TFSI, ^1^H NMR test was conducted, as shown in Figure 1b. Compared with the monomer, 1‐Butyl‐3‐vinylimidazolium bis (trifluoromethanesulfonylimine) ([BVIm]‐TFSI) (Figure S1 in Supporting Information (SI)), the ‐C‐CHN‐C‐ bond appears at 3.34 ppm, while the CH‐CH_2_ bond at 5.41 ppm disappears, suggesting that [PBVIm]‐TFSI has been successfully polymerized. The molecular weight (Mw) of as‐obtained [PBVIm]‐TFSI by gel permeation chromatography (GPC) is 1,555 g/mol.

**Figure 2:**
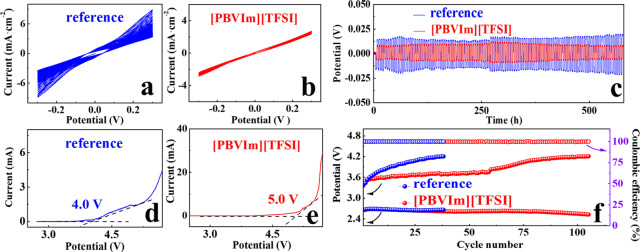
CV curves for Li|Li symmetric cells with (a) reference and (b) [PBVIm][TFSI]‐containing electrolytes at a scan rate of 3 mV⋅s^−1^ within a potential range of −0.3–0.3 V *vs*. Li/Li^+^, (c) cycling performances for Li|Li symmetric cells with reference and [PBVIm][TFSI]‐containing electrolytes at a constant current density of 0.25 mA⋅cm^−2^ under a limited capacity of 0.5 mAh⋅cm^−2^, LSV curves for cells with (d) reference and (e) [PBVIm][TFSI]‐containing electrolytes, (f) discharge‐charge profiles of cells with the different electrolytes at a constant current density of 200 mA⋅g^−1^ under the limited capacity of 500 mAh⋅g^−1^ within a potential range of 2.2–4.2 V *vs*. Li/Li^+^.

From Figure S2 in SI, it can be seen that the addition of [PBVIm]‐TFSI does not interfere with the role of I^−^ in the redox process. The influence of LiI concentration is determined in Figures S3 in SI. The proper [PBVIm]‐TFSI concentration is confirmed by comparing the cycling performances and Li^+^ migration numbers in cells with LiI and [PBVIm]‐TFSI, as shown in Figures S4 and S5 in SI. Positive charge dispersion around the imidazole ring effectively increases the cation/anion distance and reduces electrostatic interactions between ion pairs, which also helps to provide higher ionic conductivity.^[31]^ Based on these results, the eletrolyte only containing 50 mM LiI as RM is denoted as “reference” and the eletrolyte with 50 mM of LiI as RM and 100 mM of [PBVIm]‐TFSI as additive is denoted as “[PBVIm][TFSI]” in this work. To study the influence of as‐prepared electrolytes on the interface stability between Li anode and electrolyte, the plating/stripping performance for Li|Li symmetric cells with reference and [PBVIm][TFSI]‐containing electrolytes are investigated by CV tests, as seen in Figure 2. In Figure 2a, the current decreases with the cycle number for a cell with reference electrolyte. While, there is little change in the current for the one with [PBVIm][TFSI] during 50 cycles. Similarly, a Li|Li symmetric cell with [PBVIm][TFSI] exhibits a much smaller Li^+^ plating/stripping polarization than that for a reference cell, as shown in Figure 2c. These results indicate that a cell with [PBVIm][TFSI] favors a considerable interfacial stability. The oxidative stability for cells with as‐prepared electrolytes are studied by LSV tests, as shown in Figures 2d and e. The electrochemical window for [PBVIm][TFSI]‐containing electrolyte (until 5.0 V) is wider than that the reference electrolyte (until 4.0 V), which can reduce the rick for side reactions and thus promote the cyclability.[^21^, ^32^] The cyclability of cells without [PBVIm][TFSI] at a constant current density of 200 mA⋅g^−1^ under a capacity limit of 500 mAh⋅g^−1^ are also evaluated, as shown in Figure 2f, and the corresponding discharge‐charge profiles are shown in Figure S6 in SI. Compared to a span life of 38 cycles for the reference cell, the one for a cell with [PBVIm][TFSI] achieves 105 cycles, further suggesting the functional advantage of [PBVIm]‐TFSI. Furthermore, the cell with [PBVIm][TFSI] also exhibits a considerable rate performance until 60 cycles at 400 mA⋅g^−1^, as seen in Figure S7 in SI.

**Figure 3:**
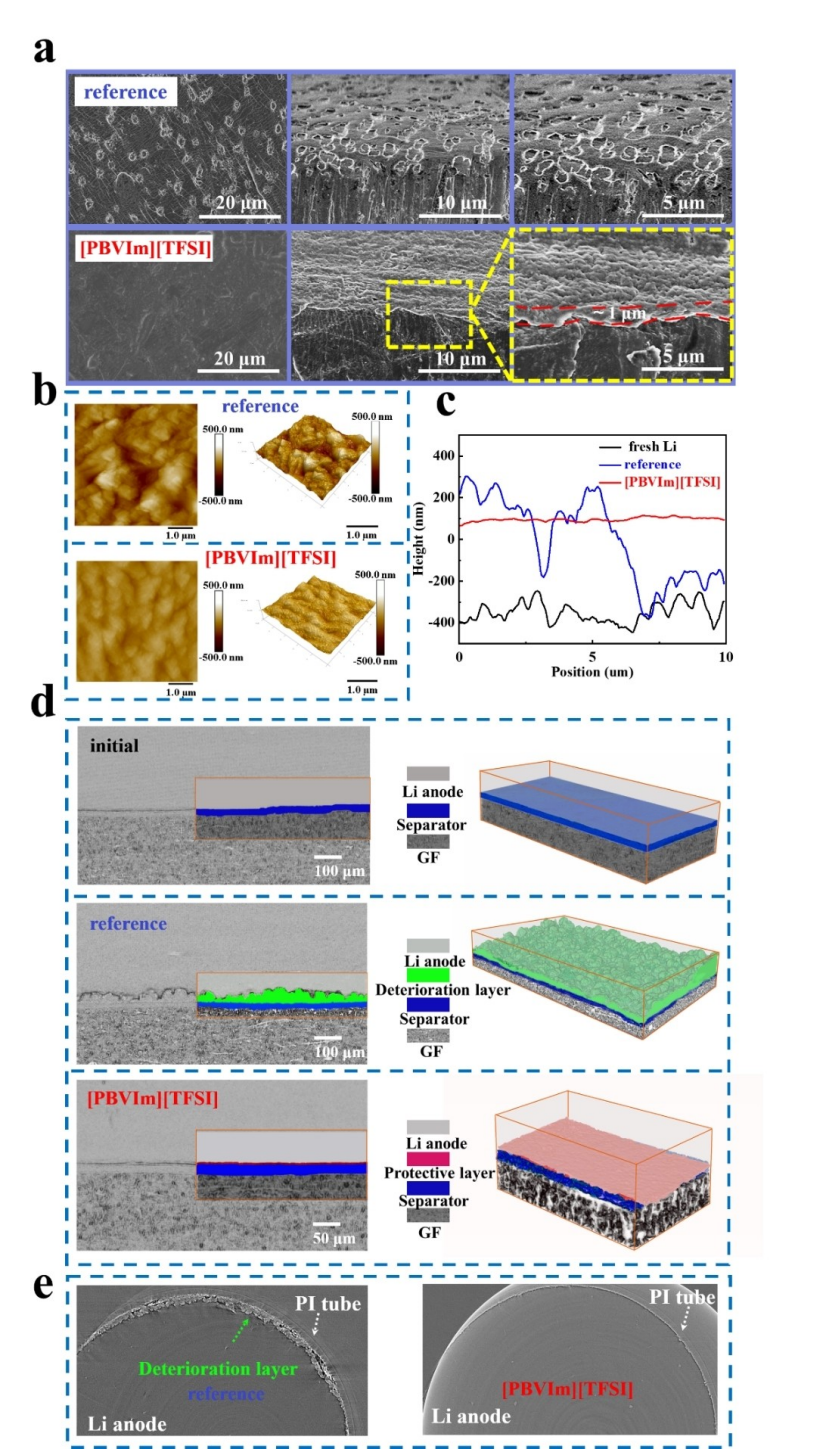
(a) SEM images (left) and the corresponding cross‐sectional views (middle and right) of Li anodes, (b) AFM images captured in an area of 10×10 μm^2^ on Li surfaces (left) and 3D images of height (right) (height scale bars are shown at the right side of each image) in cells with reference and [PBVIm][TFSI] after 5 cycles at a constant current of 200 mA⋅g^−1^ under the limited capacity of 500 mAh⋅g^−1^ within a potential range of 2.2–4.2 V *vs*. Li/Li^+^, (c) the corresponding average height curves of AFM images, (d) the cross‐sectional view and the corresponding 3D representations of Li‐O_2_ tomography cells without cycling (initial), with reference and [PBVIm][TFSI]‐containing electrolytes, (e) horizontal slice of Li‐O_2_ tomography cells with reference and [PBVIm][TFSI]‐containing electrolytes after 30 cycles at a constant current density of 0.07 mA⋅cm^−2^ under a limit capacity of 0.175 mAh⋅cm^−2^ within a potential range of 2.2–4.2 V *vs*. Li/Li^+^.

SEM images for Li anodes in cells with reference without [PBVIm][TFSI] after 5 cycles are shown in Figure 3a. Compared to the fresh Li anode (Figure S8 in SI), the Li surface in the cell with reference and without [PBVIm][TFSI] suffered from a severe corrosion, while the one in a cell with [PBVIm][TFSI] preserved a smooth surface. In a cross‐sectional view, a layer with a thickness of ~1 μm can be clearly observed in the cell with [PBVIm][TFSI]. Additionally, the [PBVIm][TFSI]‐containing cell even exhibited the smooth surface after 20 and 30 cycles (Figure S9 in SI). This provides an evidence that the addition of [PBVIm]‐TFSI can induce the *in‐situ* formation of a protective layer. Compared to the rugged Li surface cycled in the cell with reference and without [PBVIm][TFSI], the AFM image for the Li anode cycled in the cell with [PBVIm][TFSI]‐containing electrolyte exhibited smooth and uniform surfaces after 5 cycles (Figures 3b), similar to the fresh Li anode (Figure S10 in SI). The interface flutuation of the Li anode cycled in the cell with [PBVIm][TFSI] is ≦500 nm (Figure 3c). This is consistent with previous reports that cationic groups of ionic liquids can migrate to Li anode surface, forming a cationic electrostatic shielding effect, which promotes the uniform deposition of Li^+[20, 33]^. In order to investigate the interface evolution more deeply, we employed *in situ* SXCT technology for Li‐O_2_ batteries with reference and without [PBVIm][TFSI]‐containing electrolyte. A schematic of the *in‐situ* tomography model cell and an illustration of the working mechanism for SXCT can be seen in Figure S11 in SI. In Figure 3d, the tomography cell without [PBVIm][TFSI]‐containing electrolyte shows a thick complex deterioration layer on Li surface after 30 cycles, which results from an uneven Li deposition and/or Li corrosion from parasitic reactions. In contrast, a clear electrode interface and a thin layer attached to the Li anode surface can be observed in the corresponding 3D rendering image of the tomography cell with [PBVIm][TFSI] in Figure 3C. This suggests a stable interface and a good interface compatibility of the modified Li anode, which is consistent with the SEM and AFM results. Similarly, the horizontal slice of tomography cell with [PBVIm][TFSI] shows a clearer Li boundary, compared to the obvious Li corrosion in a cell without [PBVIm][TFSI] electrolyte, as seen in Figure 3e. The differences in surface and interface morphology between the cells with reference and [PBVIm][TFSI] electrolyte indicate that the protective layer can prevent redox species migrating towards to Li anode from directly contacting it, thus reducing the I_3_^−^ shuttling effect. The corresponding electrochemical curves are shown in Figure S12 in SI. This further confirms that the introduction of [PBVIm]‐TFSI plays a positive role in protecting Li anode and improving the cycling stability of Li‐O_2_ batteries.

**Figure 4:**
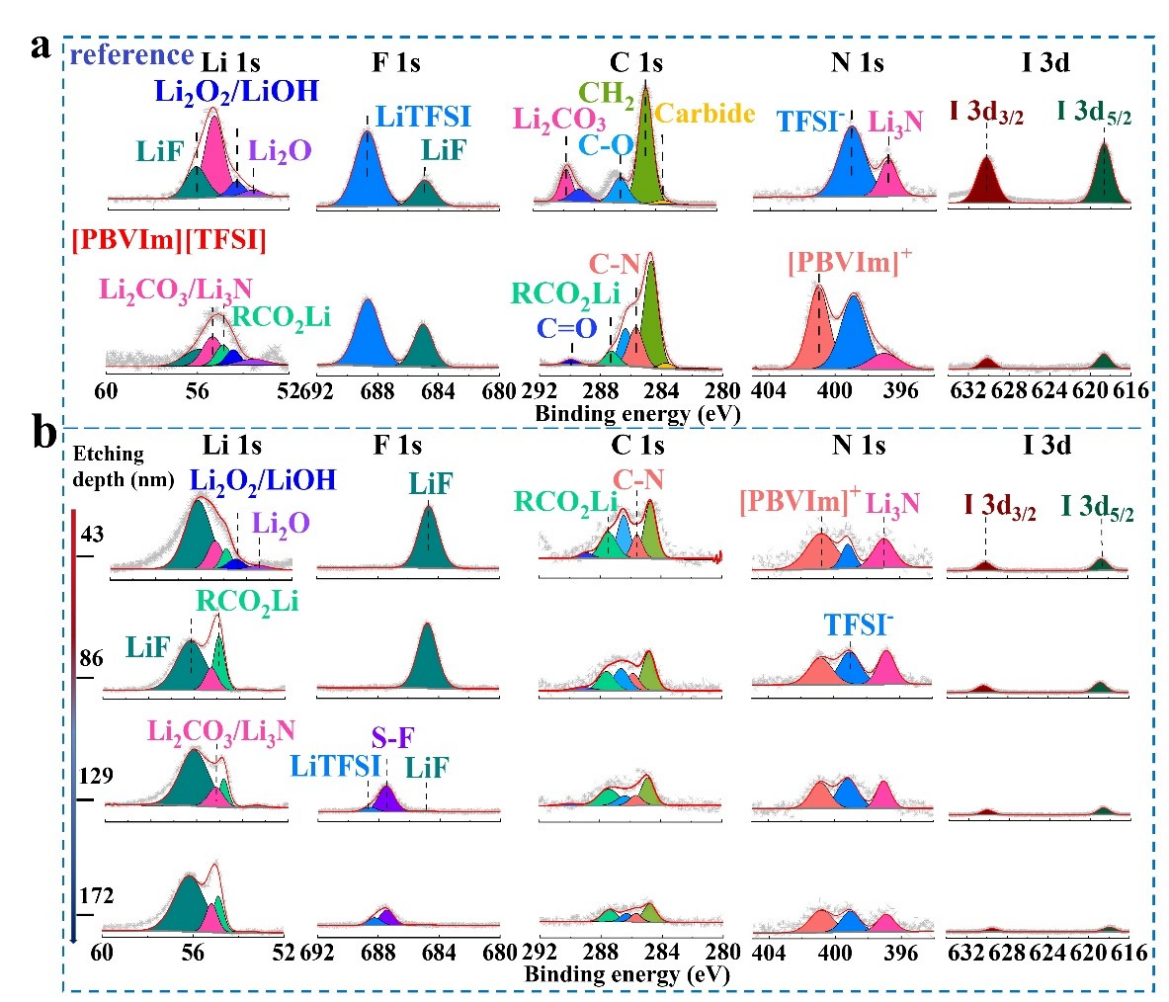
(a) XPS spectra of Li 1s, F 1s, C 1s, N 1s, and I 3d for Li anodes in cells with reference and [PBVIm][TFSI]‐containing electrolytes after 5 cycles at a constant current density of 200 mA⋅g^−1^ under a limited capacity of 500 mAh⋅g^−1^ within a potential range of 2.2–4.0 V *vs*. Li/Li^+^, (b) the corresponding spectra for Li anode with the different etching depths associated to a cell with [PBVIm][TFSI] in (a).

XPS was employed to study the chemical composition of Li surface in the cells with reference and without [PBVIm][TFSI] after 5 cycles, as shown in Figure 4a. Li 1s spectrum can be fitted into five peaks, which can be ascribed to Li_2_O (~53.3 eV), Li_2_O_2_/LiOH (~54.3 eV), RCO_2_Li (~54.8 eV), Li_2_CO_3_ (~55.3 eV), and LiF (~56.0 eV).[^34^, ^35^] The appearance of RCO_2_Li indicates that [PBVIm][TFSI] participates in the formation of the protective layer in the Li 1s and C 1s spectra.^[36]^ In general, LiF(~684.8 eV) is a side product from TFSI^−^ (~688.7 eV) decomposition, which is favorable to the formation of a stable SEI layer to protect the Li anode.^[37]^ Compared to the LiF content (18.4 at%) in the cell without [PBVIm][TFSI], the cell with [PBVIm][TFSI] shows much higher LiF content (38.9 at%), which suggests that [PBVIm]^+^ aids in the *in situ* formation of a protective layer on the Li anode derived from the TFSI anion.^[33]^ In the C 1s and N 1s spectra, C‐N (~285.6 eV) bond associated with [PBVIm]^+^ structure can only be observed in the cell with [PBVIm][TFSI],[^37^, ^38^, ^39^, ^40^] indicating that the [PBVIm]^+^ is attached on the Li surface.[^41^, ^42^] Notably, the content of I species (~0.03 at%) for the cell with [PBVIm][TFSI] is lower than that of the cell without [PBVIm][TFSI] (~0.07 at%), demonstrating that the redox shuttling is effectively suppressed by [PBVIm][TFSI] due to the *in‐situ* formation of the protective layer. To further investigate the depth profile of the constructed protective layer, Ar^+^ etching‐assisted XPS was employed. The contents of Li, F, C, N, and I with increasing etching depth are shown in Figure S13 and Table S1 in SI, and their corresponding spectra are shown in Figure 4b. As the etching depth increases, so do the S‐F ratio (~687.5 eV in F 1s spectrum) from TFSI^−^ and the Li_3_N(~398.8 eV in N 1s spectrum) content from the decomposition of imidazole in [PBVIm]^+^, confirming that the additive [PBVIm][TFSI] is involved in the SEI formation.^[43]^ The existence of [PBVIm]^+^ is observed through the whole depth profile, while the content of I species decreases from surface to bulk, futher indicating the suppression of I species shuttling. Therefore, the protective layer on the Li surface in the cell with [PBVIm][TFSI] is derived from LiTFSI decomposition and PBVIm^+^ participation, which mainly contains LiF, Li_3_N, RCO_2_Li, and Li_2_CO_3_.

**Figure 5:**
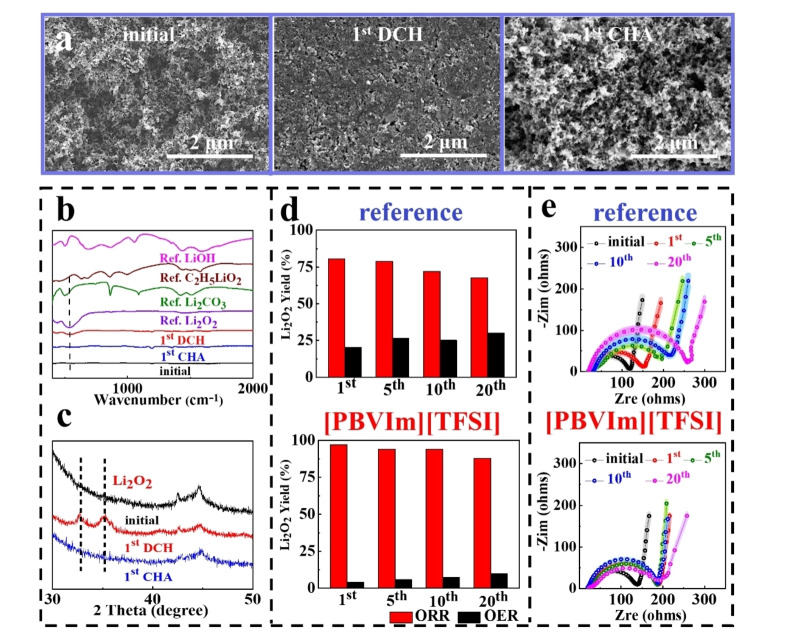
(a) SEM images, (b) FT‐IR spectra, (c) XRD patterns for O_2_ cathodes at the different electrochemical states (initial, after the 1^st^ discharge (1^st^ DCH), and after the 1^st^ recharge (1^st^ CHA)), (d) Li_2_O_2_ yields obtained from TiOSO_4_‐based titration and UV‐Vis spectroscopic analysis for O_2_ cathodes in cells with reference and [PBVIm][TFSI], (e) *in‐situ* Nyquist impedance plots in cells with reference and [PBVIm][TFSI] at the different electrochemical states using a constant current density of 200 mA⋅g^−1^ under a limit capacity of 500 mAh⋅g^−1^ within a potential range of 2.2–4.0 V *vs*. Li/Li^+^.

To confirm the [PBVIm][TFSI] effect on the electrochemical reversibility in Li‐O_2_ batteries, the O_2_ cathodes are investigated at the different electrochemical states by SEM, XRD, FT‐IR, TiOSO_4_‐based UV‐Vis titration, and EIS. In Figure 5a, SEM images show that the film‐like discharge product can be reversibly formed and decomposed in the cell with [PBVIm][TFSI]. XRD and FT‐IR results demonstrate that Li_2_O_2_ is the discharge product, which is then fully degraded during recharging process, as seen in Figures 5b and 5 c. The quantification of Li_2_O_2_ was implemented through TiOSO_4_‐based UV‐Vis titration. Detailed UV‐Vis titration data are provided in Figure S14 in SI and the standard calibration curve can be seen in Figure S15 in SI. Compared to the cell without [PBVIm][TFSI], the one with [PBVIm][TFSI] exhibits a better reversibility of Li_2_O_2_, reflected by its yield after discharge and its residual ratio after recharge, as shown in Figure 5d. The EIS results show that in initial state, the resistance of the cell without [PBVIm][TFSI] is smaller than that of the cell with [PBVIm][TFSI], as seen in Figure 5e. This could be attributed to a higher viscosity of [PBVIm][TFSI]‐containing electrolyte. However, the resistance of the cell without [PBVIm][TFSI] increases dramatically along with cycling, due to the accumulation of discharge product Li_2_O_2_ and the formation of side products from RM shuttling, and electrolyte and electrode decomposition. Note that a much more stable resistance for the cell with [PBVIm][TFSI] is observed with the increasing cycle number, suggesting a good reversibility of Li_2_O_2_ formation and degradation. The EIS is fitted according to the equivalent circuit in Figure S16 in SI, and the corresponding values are shown in Table S2 in SI.

**Figure 6:**
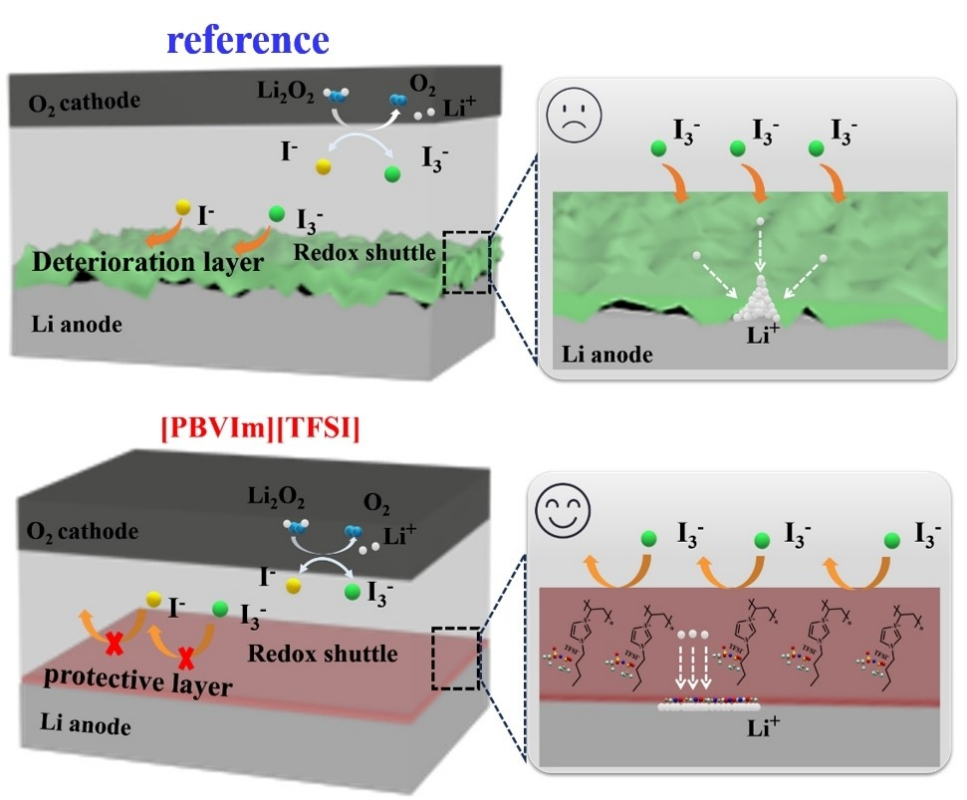
Schematic illustrations of the different mechanisms of Li‐O_2_ cells using reference and [PBVIm][TFSI]‐containing electrolytes.

As mentioned above, as‐prepared [PBVIm][TFSI] electrolyte presents several advantageous features, including an easy‐to‐operate preparation, tunable structure, good ionic conductivity, an expanded electrochemical window and enhanced stability. [PBVIm]^+^ can not only promote the uniform deposition of Li^+^ due to a cationic electrostatic shielding effect, but also facilitate the decomposition of Li salt to form a uniform protective layer on Li anode. These attributes collectively contribute to improved electrochemical performance, specifically cycling stability and rate capability. Figure 6 provides schematic illustrations depicting the distinct mechanisms involved in the cells with and without [PBVIm][TFSI]‐containing electrolytes.

## 3 Conclusions

In summary, we have developed poly((1‐Butyl‐3‐vinylimidazolium bis(trifluoromethanesulfonylimine)) ([PBVIm]‐TFSI), a functional polyionic liquid designed to tackle the critical challenge of electrode‐electrolyte interface instability in LiI‐mediate Li‐O_2_ batteries. The [PBVIm]^+^ cation migrates to the Li anode, creating a cationic electrostatic shielding effect that promotes uniform Li ion deposition. Furthermore, [PBVIm]^+^ aids in the *in situ* formation of a protective layer on the Li anode derived from the TFSI anion, effectively mitigating the shuttle effect of I_3_^−^ and significantly enhancing the cycle life of Li‐O_2_ batteries to 105 cycles. We employ scanning X‐ray computed tomography (SXCT) to investigate interfacial evolution of the protective layer in LiI‐mediate Li‐O_2_ batteries. This is complemented by atomic force microscopy (AFM) and etched X‐ray photoelectron spectroscopy (XPS) to analyze the chemical composition and to study the formation mechanisms of the protective layer. Overall, this work not only breaks through the limitation of using RM in Li‐O_2_ batteries, but also provides valuable insights into Li anode protection strategies for advanced lithium metal‐based batteries, paving the way for improved performance and longevity in energy storage technologies.

## 4 Material and Methods

### 4.1 Synthesis of [PBVIm]‐TFSI

All chemicals with analytical grade were used without further purification. The detailed synthesis process of [PBVIm]‐TFSI is shown in Figure 1a. Briefly, 1‐Butyl‐3‐vinylimidazolium bromide (1.28 g, Aladdin Reagent 97 %) and lithium bis(trifluoromethylsulphonyl)imide (LiTFSI, 5.6 g, Sigma‐Aldrich≥99.8 %) were dissolved into deionized water. The lower layer oily substance was collected after stirring for 4 h at room temperature, which was washed with deionized water for 3 times and dried in a vacuum oven at 60 °C overnight. 1‐Butyl‐3‐vinylimidazolium bis (trifluoromethanesulfonylimine) (BVIm‐TFSI) was thus obtained. The polymerization reaction was carried out at a reflux temperature of 70 °C for 12 h under Ar atmosphere, using 2 wt% of 2, 2′‐Azobis (2‐methylpropionitrile) (AIBN) as initiator and trichloromethane as solvent. The product was washed for 3 times with acetone, evaporated to remove solvents using a rotary evaporator, and then dired under vacuum at 50 °C for 24 h. [PBVIm]‐TFSI was thus prepared.

### 4.2 Electrolyte Preparation

Lithium iodide (LiI, Aladdin Reagent 99.8 %) and LiTFSI ( Sigma‐Aldrich, 99.9 %) were dried under vacuum at 120 °C for 36 h. [PBVIm]‐TFSI was dired under vacuum at 50 °C for 24 h. Tetraethylene glycol dimethyl ether (TEGDME) (Aladdin Reagent, ≥99.7 %) was dried using activated 4 Å molecular sieves (Sigma‐ Aldrich) for two weeks. The electrolytes were 50 mM LiI in 1 M LiTFSI/TEGDME with different [PBVIm]‐TFSI concentrations of 50, 100, and 150 mM. The 50 mM LiI containing electrolyte is designated as ‘reference’. All the procedures were carried out in an Ar‐filled glove box (Mikrouna, both H_2_O and O_2_<0.1 ppm).

### 4.3 O_2_ Cathode Preparation

The preparation for O_2_ cathode followed the reference.^[44]^ Super P carbon (SP, lithium battery grade, TIMCAL) and polyvinylidene fluoride (PVDF, Solef@5130, Solvay) were dierd under vacuum at 120 °C for 24 h and then cooled naturally to room temperature for futher using. SP and PVDF in a weight ratio of 9 : 1 were mixed and dispersed in N‐methyl‐2‐pyrrolidone (NMP, Aladdin Reagent, 99.5 %) to make a slurry. The slurry was then cast onto a carbon paper substrate (CeTech Co., Ltd.) with a diameter of 12 mm, which was dierd in a vacuum oven at 120 °C overnight. After the NMP evaporation, the cathode was thus obtained. The total mass loading for the cathode was 0.3±0.05 mg.

### 4.4 Cell Assembly

All cell assembly processes were carried out in an Ar‐filled glove box (Mikrouna, both H_2_O and O_2_<0.1 ppm). The Li‐O_2_ battery employed a 2025 type coin cell with seven holes of 2 mm diameter in the cathode case, which consisted of a Li foil anode (China Energy Lithium Co., Ltd.), a single‐layer glass fiber separator (GF/D, Whatman) immersed in as‐prepared electrolyte, and an O_2_ cathode. Li|Li symmetric cells were assembled with a single‐layer glass fiber separator immersed in as‐prepared electrolytes between the two Li electrodes in 2025 type coin‐cell.

All tomography cells were assembled using special customized molds with stainless steel screws on both sides and poly(ether‐ether‐ketone) (PEEK) material in the middle section for X‐rays transmission (Fig. S11), which followed in previous report.[^45^, ^46^] In addition, the same electrode materials were used as ordinary Li‐O_2_ cells to study the interface evolution. All tomography cells were assembled in a glove box. During a tomography cell assembly, a 2.5 mm diameter Li anode was placed on top of a stainless‐steel screw, followed by a 3.0 mm diameter single‐layer glass fiber (GF/D, Whatman) separator, a 3.0 mm diameter porous O_2_ cathode and 3.0 mm diameter nickel foam. The steel screw with a drilling hole was in contact with the buffered nickel foam. All tomography cells were sealed in a glove box after installation, and then were connected to an oxygen tube after being taken out. To ensure complete oxygen absorption, all cells rested for 4 h before electrochemical cycling.

### 4.5 Electrochemical Tests

All electrochemical measurements were carried out in an ultra‐high purified O_2_‐filled glovebox (H_2_O<0.1 ppm) using LAND battery testing system (CTA2001A, Wuhan Land Electronic Co., Ltd.). CV tests were performed on a potentiostat (PARSTAT 4000) in a three‐electrode system, consisting of a glass carbon electrode as a working electrode (φ =5 mm, Gaoss Union Technology Co., Ltd.), a Pt wire as a counter electrode (Pt0537, Gaoss Union Technology Co., Ltd.), and a Ag/Ag^+^ non‐aqueous reference electrode (BASMF2062)^[47]^ at a scan rate of 50 mV⋅s^−1^ within a potential range of −0.8–1.8 V *vs*. Ag/Ag^+^. The electrochemical oxidative stability of as‐prepared electrolytes was tested by a potentiostat (PARSTAT 4000). Linear sweep voltammetry (LSV) test was carried out using a cell with a Li foil | a glass fiber filter immersed in as‐prepared electrolytes | a stainless steel electrode at a scan rate of 0.1 mV⋅ s^−1^ within a potential range from open circuit voltage (OCV) to 6.0 V *vs*. Li/Li^+^. Galvanostatic measurements were performed using a LAND battery testing system (CTA2001 A, Wuhan Land Electronic Co., Ltd.). The cyclability for Li‐O_2_ cells was studied at a constant current density of 200 mA⋅g^−1^ based on carbon weight in the O_2_ cathode under specific capacity limitation of 500 mAh⋅g^−1^ within a potential range from 2.2 to 4.2 V *vs*. Li/Li^+^. All current and capacity calculations were based on carbon weight in the O_2_ cathode. All tomography cells were cycled at a constant current density of 0.07 mA⋅cm^−2^ under a limit capacity of 0.175 mAh⋅cm^2^ within a potential range of 2.2–4.2 V vs. Li/Li^+^.

Electrochemical impedance spectroscopy (EIS) test was carried out using an amplitude of 10 mV and a frequency range of 10^6^–10^−2^ Hz. Li^+^ transfer number (t_Li_^+^) was measured on the Li|Li symmetric coin cell by AC impedance and DC polarization. The Bruce‐Vincent‐Evans formula can be used to calculate the t_Li_^+^ of the electrolyte as following:

$$t_{{Li}^{+}}=\frac{I_{s}\left(\Delta V-I_{0}R_{0}\right)}{I_{0}\left(\Delta V-I_{s}R_{s}\right)}$$

where I_0_, I_S_, R_0_, and R_S_ are the initial current, the steady‐state current, the initial interfacial resistance, and the steady‐state interfacial resistance, respectively.

### 4.6 SXCT Test

The synchrotron X‐ray tomography tests were carried out at the BL13HB Beamline at Shanghai Synchrotron Radiation Facility (SSRF) of China. Figure S11 in SI illustrates the designed tomography cell and the employed beamline configuration. The synchrotron beam was monochromatized to 20 keV using a double crystal monochromator (DMM), utilizing X‐ray CCD detector with a spatial resolution of 1.625 μm/pixel. The image dimension field was 3.328×3.328 mm, and the achieved spatial resolution was 0.8 μm. One synchrotron X‐ray tomography was performed, while the cell was rotated 180°, and 1024 projections were recorded with an exposure time of 100 ms per projection. Afterwards, the filtered back‐projection was used for the final reconstruction. Avizo Studio 2019.1 was used for 3D rendering of the tomography cell. This part of the synchrotron radiation test can refer to the previous work.[^48^, ^49^]

### 4.7 Characterization

The structure of as‐prepared [PBVIm]‐TFSI was identified by ^1^H NMR on a Bruker AVANCE 600 MHz spectrometer. Gel permeation chromatography (GPC) was performed to measure molecular weight of [PBVIm]‐TFSI using DMF as eluent (1.0 mL⋅min^−1^) at 40 °C on an Agilent 1260 Infinity Series instrument. The morphology and element analysis for electrodes at different electrochemical states were performed by scanning electron microscope (SEM) coupled with energy dispersive spectroscopy (Hitachi S‐5500 with EDS AMETEK). AFM (Asylum Cypher ES) was employed to evaluate the surface morphology for Li anodes at different electrochemical states. XPS measurement was carried out to investigate the surface composition and chemical states for Li anode at different electrochemical states with an ESZALB 250XL spectrometer using Al Kα radiation (hν=1486.7 eV) and an emission angle of 90^○^. All binding energies were calibrated using C 1s peak assigned to C‐C bond at 284.8 eV. The chemical composition of the protective layer on Li anode was characterized by XPS depth profile measurement using a 20 eV Ar^+^ beam with a single etching time of 200 s at a sputtering rate of 0.167 nm⋅s^−1^ (using the etching rate of SiO_2_ as a reference). The XRD analysis of O_2_ cathode was performed using a Rigaku‐Dmax 2500 diffractometer with Cu Kα radiation (λ=1.5406 Å), operating at 40 kV and 30 mA. Infrared spectroscopy was conducted on a Nicolet 6700 FT‐IR spectrometer for O_2_ cathodes. The quantification of Li_2_O_2_ formation and decomposition was achieved *via* a UV‐Vis spectrometry (Cary60, VARIAN) using a TiOSO_4_‐based Li_2_O_2_ titration method.[^50^, ^51^] The cathodes and GF/D separators were immersed in 10 mL of TiOSO₄ solution (Sigma‐Aldrich, 99.99 %) containing 4.5–5.5 wt% Ti in a 15 wt% dilute sulfuric acid matrix. Li_2_O_2_ reacted with the H_2_O in solution to generate H_2_O_2_, which subsequently reacted with Ti^4+^ to form the yellowish [Ti(O_2_)]^2+^ complex. This complex was identified by a characteristic absorption peak at λ_max_=409 nm in the UV‐Vis spectrum.^[52]^ The amount of Li_2_O_2_ was quantified through comparison with a calibration curve obtained using a range of known amounts of commercial Li_2_O_2_.

## Conflict of Interests

The authors declare no conflict of interest.

5

## Supporting information

As a service to our authors and readers, this journal provides supporting information supplied by the authors. Such materials are peer reviewed and may be re‐organized for online delivery, but are not copy‐edited or typeset. Technical support issues arising from supporting information (other than missing files) should be addressed to the authors.

Supporting Information

## Data Availability

The data that support the findings of this study are available in the supplementary material of this article.
