# Supplementary material for: Polymeric Ionic Liquid‐Enabled In Situ Protection of Li Anodes for High‐Performance Li‐O2 Batteries
Source: ChemSusChem. 2024 Nov 28;18(7):e202402102. doi: 10.1002/cssc.202402102 (PMC11960590; doi:10.1002/cssc.202402102)
Supplement: Supplementary file 1 — Supporting Information [file CSSC-18-e202402102-s001.pdf]

ChemSusChem

Supporting Information

Polymeric Ionic Liquid-Enabled *In Situ* Protection of Li Anodes for High-Performance Li-O_2 Batteries

Dan Li, Qian Chen, Rui Li, Yaolin Hou, Yulong Liu, Haiming Xie, Jia Liu,* and Jiefang Zhu*

Supporting Information

Polymeric Ionic Liquid-Enabled *In Situ* Protection of Li Anodes for High-Performance Li-O_2 Batteries

Dan Li[a,b]*, Qian Chen*[c]*, Rui Li*[a]*, Yaolin Hou*[a]*, Yulong Liu*[a]*, Haiming Xie*[a]*, Jia Liu*[*a]*, Jiefang Zhu*[*b,d]

[a]Nation & Local United Engineering Laboratory for Power Batteries, Faculty of Chemistry, Northeast Normal University, Changchun, Jilin, 130024, China.

[b]Department of Chemistry – Ångström Laboratory, Uppsala University, SE-751 21, Sweden.

[c]Jilin Province Dongchi New Energy Technology Co., Ltd. Changchun, Jilin, 130000, China.

[d]The Key Laboratory for Ultrafine Materials of The Ministry of Education, East China University of Science and Technology, Shanghai 200237, China.

E-mail: liuj645@nenu.edu.cn; jiefang.zhu@kemi.uu.se.

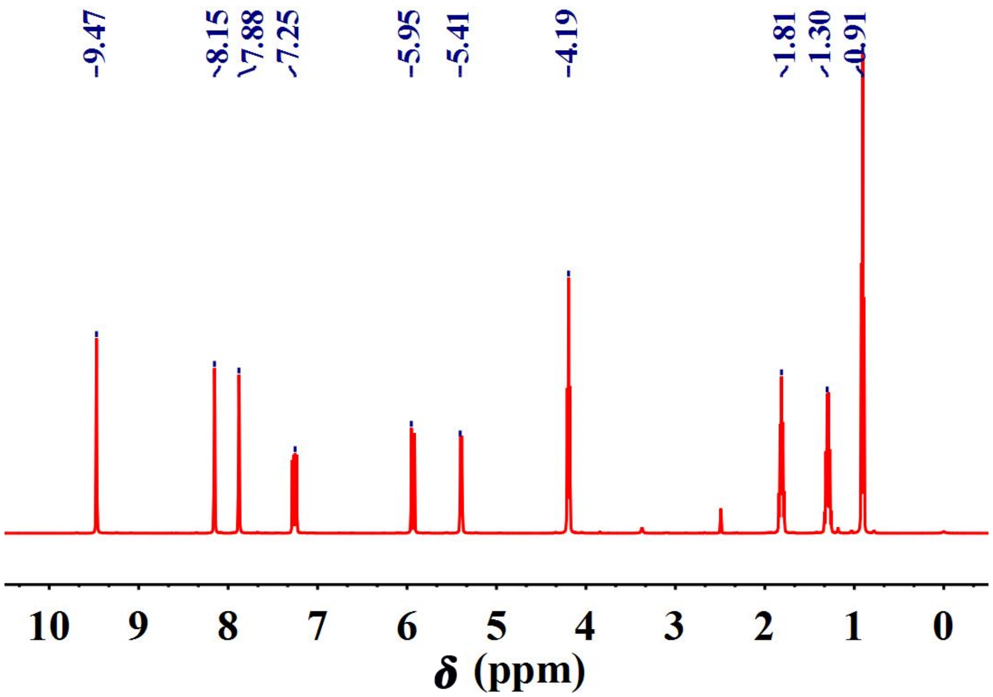

Figure S1. ^{1}H NMR spectrum of [BVIm]-TFSI complex in DMSO-d6.

The ^{1}H NMR spectrum analysis is as follows:

[BVIm]-TFSI: ^{1}H (500 MHz, DMSO-d6) δ (ppm): 9.47(s, 1H), 8.18 (s, 1H), 7.88 (s, 1H), 7.25 (t, 1H), 5.95(d, 1H), 5.41 (d, 1H), 4.19 (t, 2H), 1.84 (m, 2H), 1.31 (m, 2H), 0.91(m, 3H).

DMSO-d6: ^{1}H (500 MHz, DMSO-d6) δ (ppm): 2.54

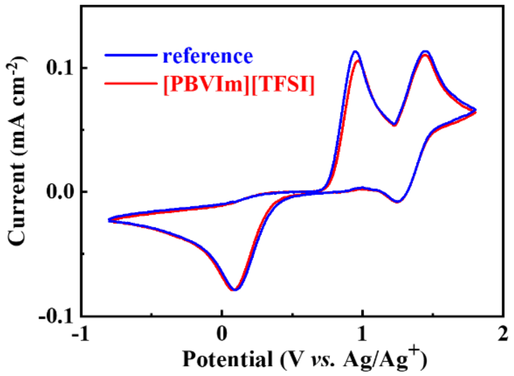

Figure S2. CV curves for three-electrode systems using reference and [PBVIm][TFSI]-containing electrolytes in an Ar atmosphere at a scan rate of 50 mV·s^{-1} within a potential range of -0.8-1.8 V *vs.* Ag/Ag^{+}.

Two pairs of oxidation/reduction peaks corresponding to the redox potential of I^-/$I_3^-$and I_3^-/I_2 were observed at 0.95/0.09 V and 1.45/1.25 V, respectively. No significant differences in the redox potential position, the current peak intensity and shape were observed between the reference and [PBVIm][TFSI]-containing electrolyte, indicating that the introduction of [PBVIm]-TFSI does not affect the role of LiI in redox processes.

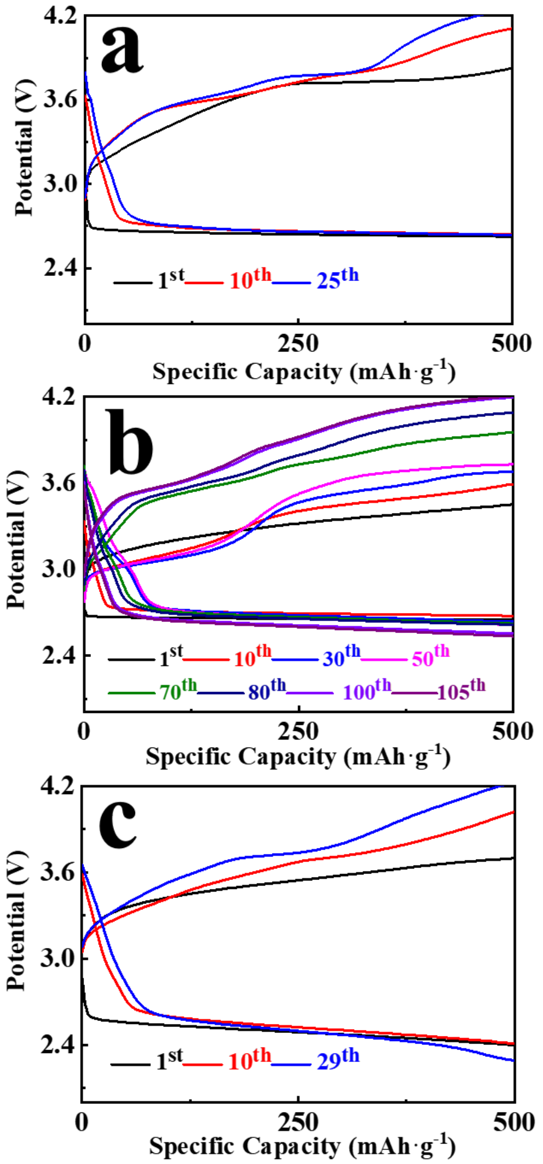

Figure S3. The cycling responses of cells with (a) 25 mM LiI and 100 mM [PBVIm]-TFSI, (b) 50 mM LiI and 100 mM [PBVIm]-TFSI, (c) 100 mM LiI and 100 mM [PBVIm]-TFSI-containing electrolytes at a constant current density of 200 mA·g^{-1} under a limited capacity of 500 mAh·g^{-1} within a potential range of 2.2-4.2 V *vs.* Li/Li^{+}.

In order to investigate the effect of different concentrations of LiI on the cycling performance of Li-O_2 batteries with [PBVIm]-TFSI-containing electrolyte, we added 25 mM, 50 mM and 100 mM LiI to the electrolyte containing 100 mM [PBVIm]-TFSI. A cell with 25 mM LiI showed 25 cycles, a cell with 50 mM LiI showed 105 cycles and a cell with 29 cycles at 100 mM LiI. Based on these results, we chose the electrolyte containing 50 mM LiI for the following test.

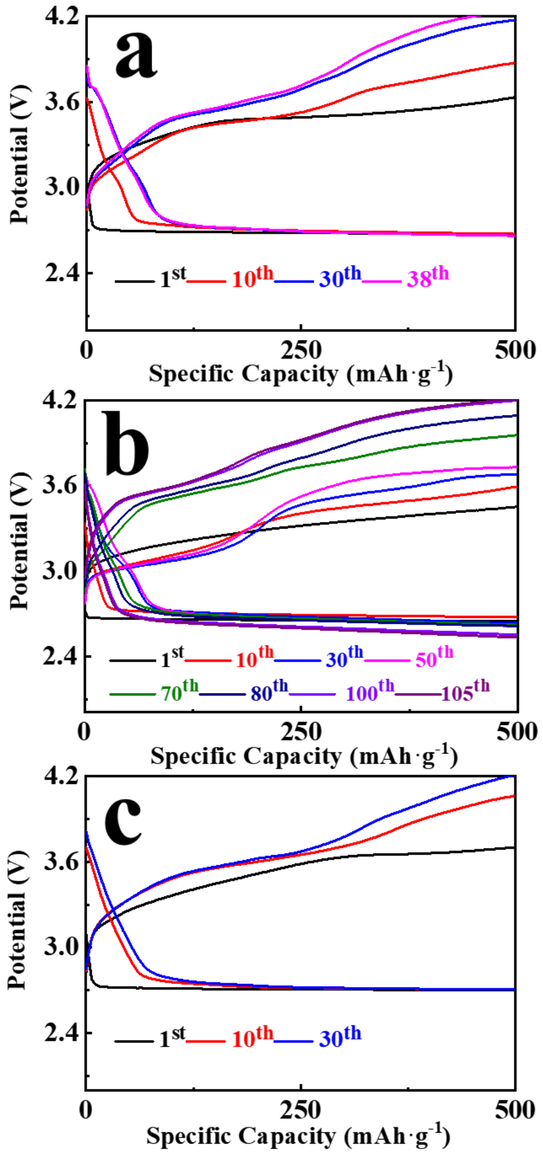

Figure S4. The cycling responses for cells with (a) 50 mM LiI and 50 mM [PBVIm]-TFSI, (b) 50 mM LiI and 100 mM [PBVIm]-TFSI, (c) 50 mM LiI and 150 mM [PBVIm]-TFSI-containing electrolytes at a constant current density of 200 mA·g^{-1} under a limited capacity of 500 mAh·g^{-1} within a potential range of 2.2-4.2 V *vs.* Li/Li^{+}.

In order to investigate the effect of different concentrations of [PBVIm]-TFSI on the cycling performance of Li-O_2 batteries with LiI, we added 50 mM, 100 mM and 150 mM [PBVIm]-TFSI to the electrolyte containing 50 mM LiI. A cell with 50 mM [PBVIm]-TFSI showed 38 cycles, a cell with 100 mM [PBVIm]-TFSI showed 105 cycles and a cell with 150 mM [PBVIm]-TFSI showed 30 cycles. Therefore, we chose the cell containing 50 mM LiI and 100 mM [PBVIm]-TFSI as representative sample, denoted as "[PBVIm][TFSI]".

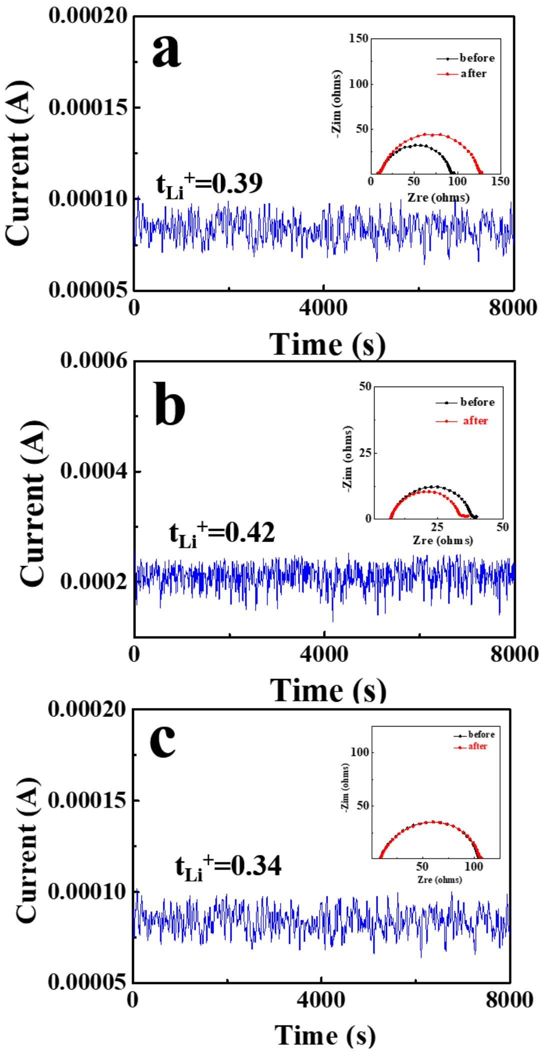

Figure S5. Current *vs.* Time plots following a DC polarization of 10 mV of the Li|Li symmetric cells with (a) 50 mM LiI and 50 mM [PBVIm]-TFSI, (b) 50 mM LiI and 100 mM [PBVIm]-TFSI, (c) 50 mM LiI and 150 mM [PBVIm]-TFSI-containing electrolytes. Inset: EIS plots before and after polarization.

Li^+ transfer numbers in the cells containing LiI and [PBVIm]-TFSI were evaluated by using Li|Li symmetric cells. The cell assembly involved using Li chips with a diameter of 13 mm for both cathode and anode, a 16 mm diameter GF/D separator immersed with 120 µL of electrolyte. The Li^+ transfer numbers was 0.39 for a cell containing 50 mM LiI and 50 mM [PBVIm]-TFSI, 0.42 for a call with 50 mM LiI and 100 mM [PBVIm]-TFSI and 0.34 for a cell with 50 mM LiI and 150 mM [PBVIm]-TFSI.

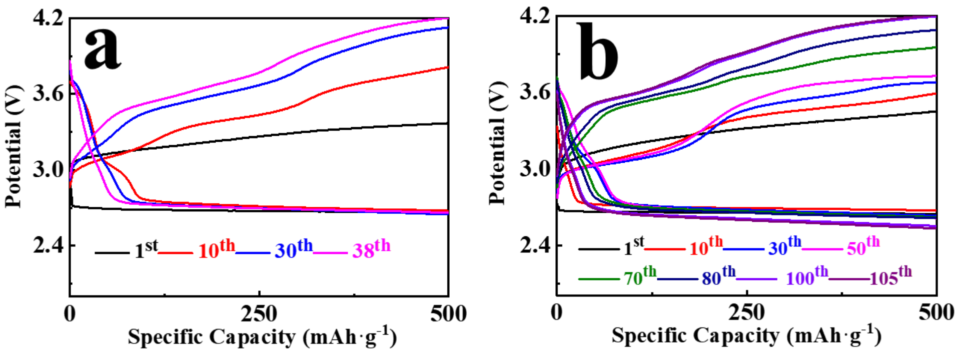

Figure S6. The cycling responses of Li-O_2 batteries with the (a) reference and (b) [PBVIm][TFSI]-containing electrolytes at a constant current density of 200 mA·g^{-1} under a limited capacity of 500 mAh·g^{-1} within a potential range of 2.2-4.2 V *vs.* Li/Li^+.

The cycling performance of the cells with and without [PBVIm][TFSI]-containing electrolytes were measured. Compared to 38 cycles of a cell with the reference electrolyte, the one in a cell with [PBVIm][TFSI]-containing electrolyte achieved 105 cycles. This confirms that the introduction of [PBVIm][TFSI] into LiI-based Li-O_2 batteries can prolong cycle life.

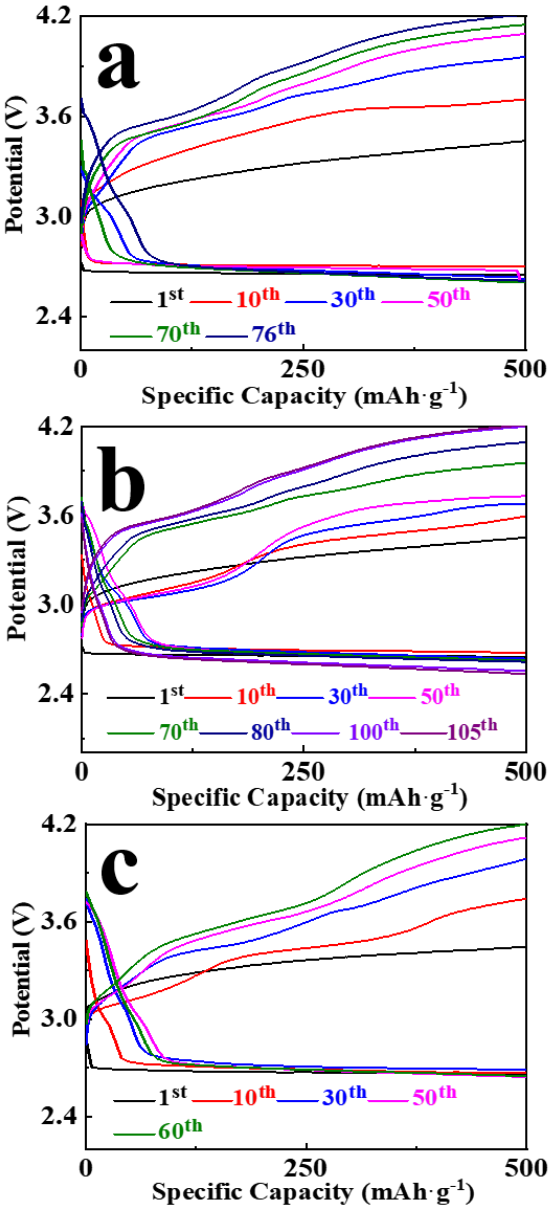

Figure S7. The discharge-charge profiles for Li-O_2 batteris with [PBVIm][TFSI] at the various constant current densities of (a) 100, (b) 200, and (c) 400 mA·g^{-1} under a limited capacity of 500 mAh·g^{-1} within a potential range of 2.2-4.2 V *vs.* Li/Li^+.

In order to further investigate the cycling performance of [PBVIm][TFSI]-containing electrolyte, galvanostatic discharge-charge measurements at 100, 200, and 400 mA·g^{-1} were performed. The discharge-charge cycling was carried out for 76 cycles with the current 100 mA·g^{-1},105 cycles with the current 200 mA·g^{-1} and 60 cycles with the current 400 mA·g^{-1} under a limited capacity of 500 mAh·g^{-1}. Those results indicate that the cell containing [PBVIm][TFSI] exhibits good cyclability.

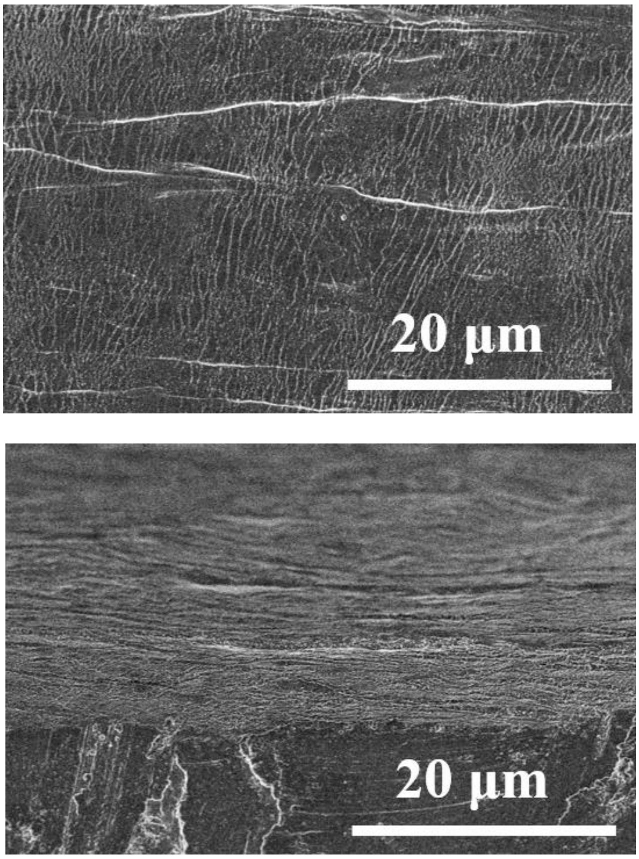

Figure S8. SEM image (top) and the corresponding cross-sectional view (bottom) for a fresh Li anode.

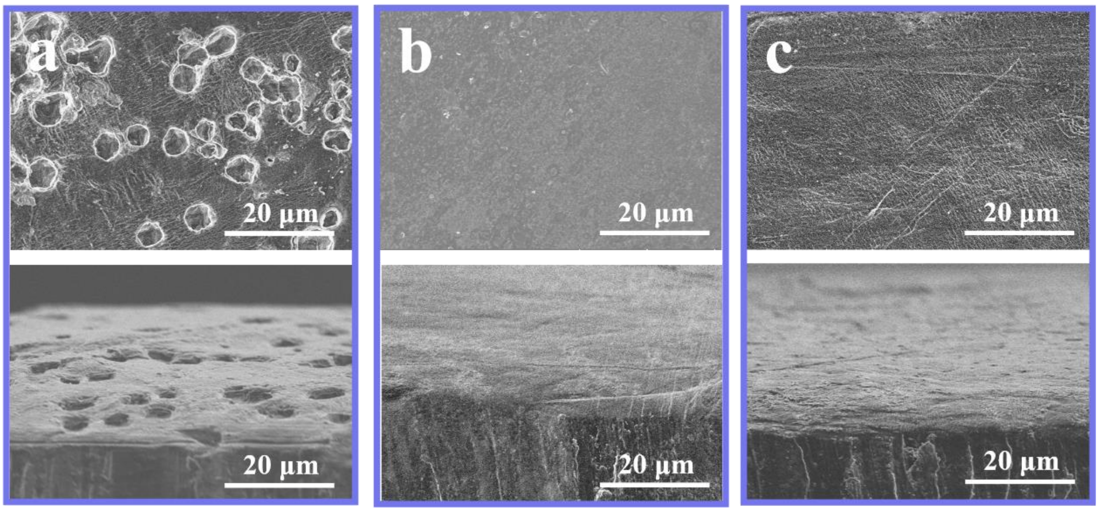

Figure S9. SEM images (top) and the corresponding cross-sectional views (bottom) for Li anodes in cells with the (a) reference and (b) [PBVIm][TFSI]-containing electrolyte after 20 cycles; (c) Li anodes in a cell with [PBVIm][TFSI] after 30 cycles at a constant current density of 200 $mA \cdot g^{-1}$ under a limited capacity of 500 $mAh \cdot g^{-1}$ within a potential range of 2.2-4.2 V *vs.* Li/Li^+.

Figure S9 shows that the Li anode surface in the cell with the reference electrolyte exhibits significant corrosion and an uneven morphology after 20 cycles. In contrast, a more uniform and dense Li surface morphology is observed in the cell with [PBVIm][TFSI]-containing electrolyte, remaining smooth even after 30 cycles. These SEM images demonstrate that the addition of [PBVIm]-TFSI effectively inhibits the I_3^- shuttling effect.

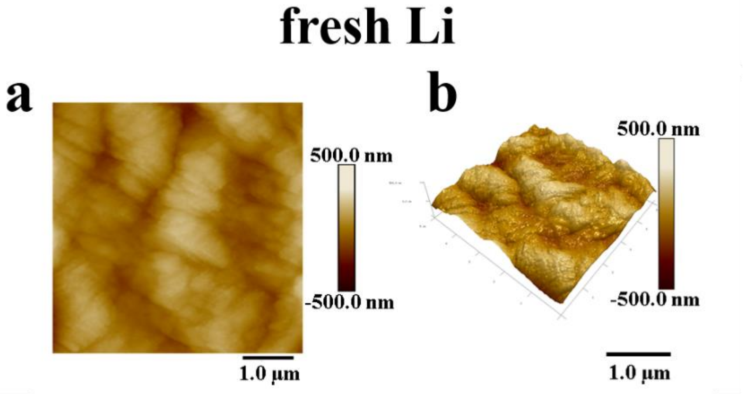

Figure S10. (a) The surface and (b) 3D reconstruction images for a fresh Li anode by AFM.

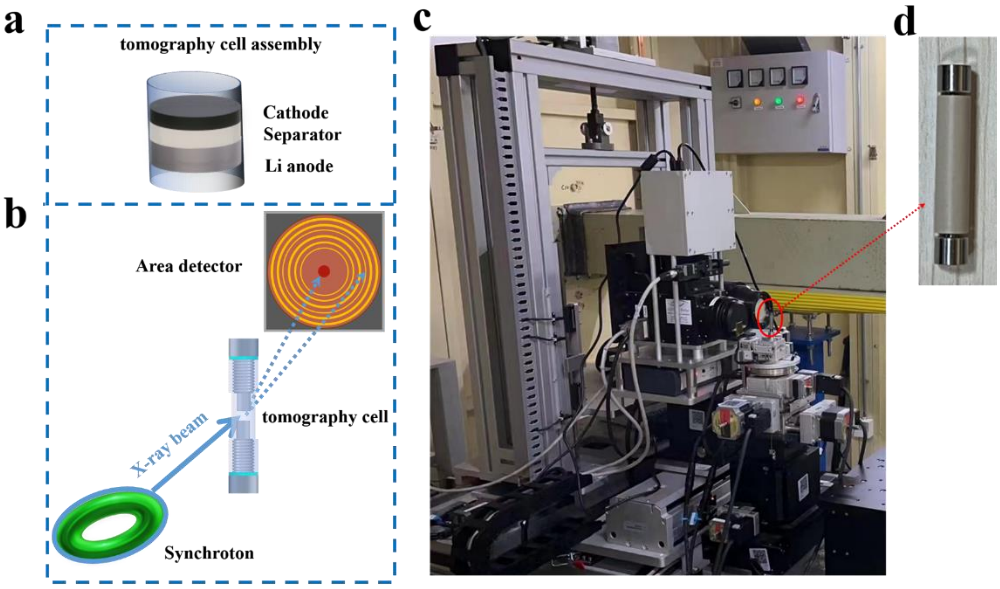

Figure S11. Schematic illustrations of (a) the tomography cell and (b) the synchrotron X-ray tomography; the photos of (c) the SXCT beamline station BL13HB at Shanghai Synchrotron Radiation Facility (SSRF) of China and (d) Li-O_2 tomography cell.

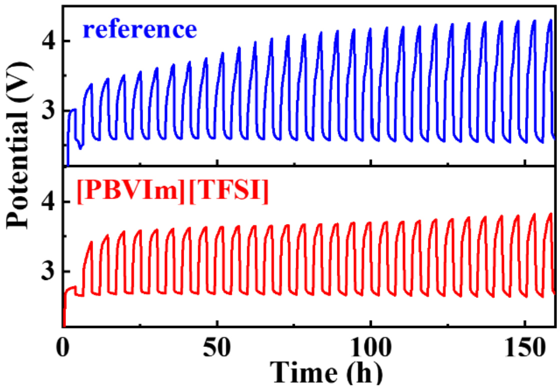

Figure S12. Electrochemical characterizations of the investigated Li-O_2 tomography cells with the reference and [PBVIm][TFSI]-containing electrolytes at a constant current density of 0.07 mA·cm^{-2} under a limit capacity of 0.175 mAh·cm^{-2} within a potential range of 2.2-4.2 V *vs.* Li/Li^+ corresponding to **Figure 3d** in the main manuscript.

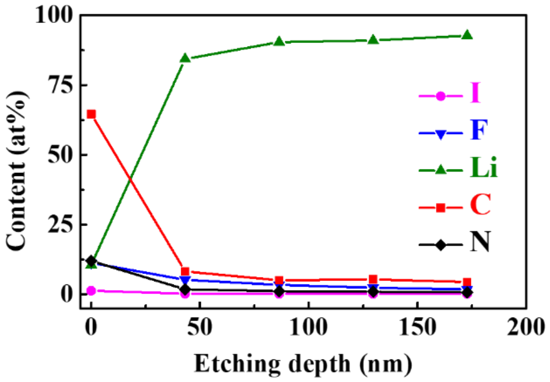

Figure S13. The content evolutions for the different elements of Li anode with the different etching depths.

Table S1. The elements content analysis of a Li anode with the different etching depths in Li-O_2 batteries with [PBVIm][TFSI] after 5 cycles at a constant current density of 200 mA·g^{-1} under a limited capacity of 500 mAh·g^{-1} within a potential range of 2.2-4.2 V *vs.* Li/Li^+.

Content (at%) / Etching depth(nm)	Li	F	C	N	I
0	11.96	11.33	64.65	12.03	0.03
43.2	84.61	5.26	8.27	1.84	0.02
86.4	90.27	3.45	5.08	1.18	0.02
129.6	91.19	2.40	5.38	1.03	0.01
172.8	92.89	1.86	4.48	0.76	0.01

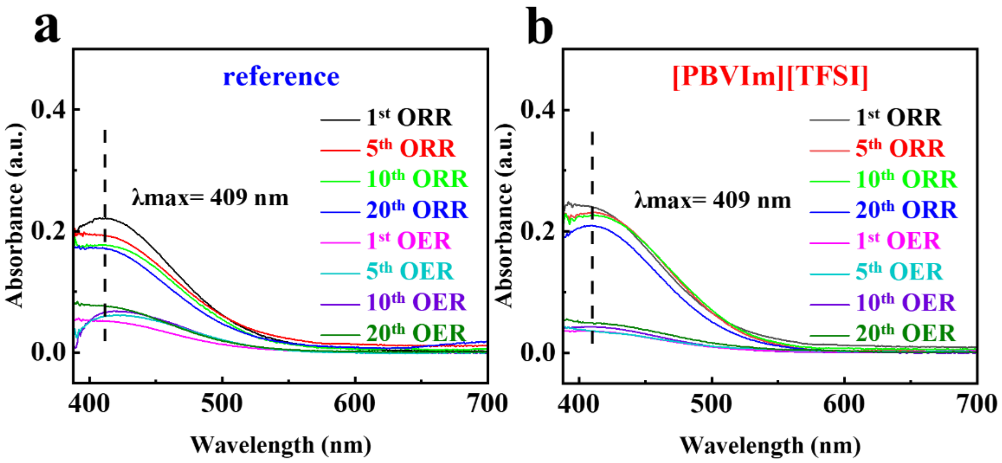

Figure S14. UV-vis absorption spectra of the titration solutions with the cathodes in the Li-O_2 batteries with (a) the reference and (b) [PBVIm][TFSI]-containing electrolytes.

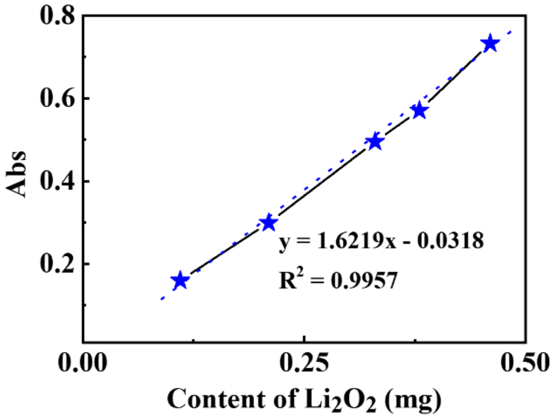

Figure S15. The standard calibration curve for Li_2O_2 obtained *via* a $TiOSO_4$-based Li_2O_2 titration method using a UV-Vis spectrometry.

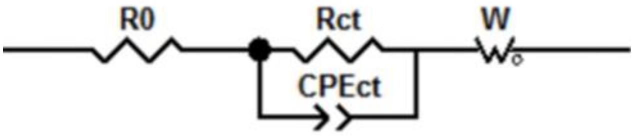

Figure S16. The equivalent circuit corresponding to *in situ* Nyquist plots of Li-O_2 cells with the reference and [PBVIm][TFSI]-containing electrolytes in **Figure 5e** of the main text.

Table S2. The fitting values of R_0, R_{ct}, and R_{total} for the cells with the reference and [PBVIm][TFSI]-containing electrolytes at different electrochemical states with a constant current density of 200 mA·g^{-1} under a limit capacity of 500 mAh·g^{-1} within a potential range of 2.2-4.2 V *vs* Li/Li^+.

		reference	[PBVIm][TFSI]
R_o(Ω)	initial	21.12	22.75
	after 1 cycle	21.90	23.28
	after 5 cycles	24.32	28.92
	after 10 cycles	22.28	21.75
	after 20 cycles	21.31	23.84
R_{ct}(Ω)	initial	92.73	113.67
	after 1 cycle	127.82	162.44
	after 5 cycles	172.30	157.47
	after 10 cycles	191.64	164.64
	after 20 cycles	236.27	166.46
R_{total}(Ω)	initial	113.85	136.42
	after 1 cycle	149.72	185.72
	after 5 cycles	196.62	186.39
	after 10 cycles	213.92	186.60
	after 20 cycles	257.58	190.30
